# Decoding the Knacks of Ellagitannin Lead Compounds to Treat Nonalcoholic Fatty Liver Disease using Computer-aided Drug Designing

**DOI:** 10.2174/0115734099325555240927054614

**Published:** 2024-10-07

**Authors:** Hina Shahid, Muhammad Ibrahim, Wadi B. Alonazi, Zhanyou Chi

**Affiliations:** 1 MOE Key Laboratory of Bio-Intelligent Manufacturing, School of Bioengineering, Dalian University of Technology, Dalian, Liaoning, China;; 2 State Key Laboratory of Rice Biology and Breeding, Key Laboratory of Molecular Biology of Crop Pathogens and Insects, Institute of Biotechnology, Zhejiang University, Hangzhou, 310058, China;; 3 Health Administration Department, College of Business Administration, King Saud University, Riyadh, Saudi Arabia

**Keywords:** Non-alcoholic fatty liver disease, virtual screening, drug designing, molecular modeling, dynamics simulations, computational analysis

## Abstract

**Background:**

The prevalence of nonalcoholic fatty liver disease (NAFLD) is increasing globally, impacting individuals in Western nations and rapid growing in Asian countries due to sedentary lifestyles; thus, NAFLD has emerged as a significant worldwide health concern. Presently, lifestyle changes represent the primary approach to managing NAFLD.

**Methods:**

This research aimed to identify the potential drug targets for treating NAFLD through comprehensive *in silico* computational analysis. These include the prediction of the three-dimensional structure of the protein, the prediction of inhibitors by PubChem and ZINC, molecular docking by Autodcok, pharmacophore modeling, molecular dynamics simulation by the OPLS_2005 force field, and the orthorhombic box solvent model Intermolecular Interaction Potential 3 Points Transferable to the selected compound. The toxicity of the lead compounds was analyzed through AdmetSAR software.

**Results:**

The protein associated with the PNPLA3 gene, whose overall three-dimensional structure was 95% accurate, were retrieved following inhibitor selection *via* PubChem and ZINC. Among the selected inhibitors and docked compounds with ID 10033935 (ellagitannin) showed a minimum E-Score of -17.266. In docking and pharmacophore modeling the compound ellagitannin shows promise as a potenstial drug candidate. Moreover, the molecular dynamics and structural stability of the protein-ligand complex were evaluated with several metrics such as as root mean square fluctuation and root mean square deviation and resulted in the stability not only of PNPLA3-10033935 (ellagitannin) but also of compound PNPLA3-71448940 and PNPLA3-5748394 complexed proteins at 400 ns with very slight variation.

**Conclusion:**

Overall, ellagitannin was identified as the best druggable target with the best therapeutics profile. The findings of our study can pave the way for the development of a new drug against NALFD.

## 1 INTRODUCTION

With an estimated 30% global prevalence, nonalcoholic fatty liver disease (NAFLD) is one of the most common causes of chronic liver disorders not only in adults but also in children and adolescents, with a 7.4% global prevalence. The incidence of this disease is increasing nearly simultaneously with that of obesity and type 2 diabetes (T2D) [1-3]. The progressive presence of NAFLD or NASH can result in liver-related mortality and is linked to decreased health-related quality of life, declining worker productivity, and poor healthcare resource utilization [4]. Lifestyle modification remains the cornerstone of current NAFLD and NASH treatment, and several new drug regimens are under development. The support of modern NAFLD and NASH treatment is still a lifestyle change; however, several novel medication treatments are being established [5, 6]. Despite a substantial increase in the incidence of NAFLD over the past three decades, awareness about NAFLD and its symptoms remains deficient, and it is not officially recognized by the World Health Organization as a significant noncommunicable disease [7-9].

Despite its high frequency, NAFLD often develops without noticeable symptoms and silently. Moreover, alcoholic liver disease has become the most common chronic liver disease in Western countries [10]. The development and growth of NAFLD are influenced by many factors such as environmental, behavioral factors, and a multitude of genetic factors that contribute to the wide range of liver abnormalities. When the disease is untreated, fibrosis and cirrhosis can occur in approximately 20% of individuals with NASH [11, 12] and a robust association between obesity, insulin resistance, and NAFLD has been reported, all of which are components of metabolic syndrome that leads to diabetes, cardiovascular diseases, and stroke [13, 14]. To comprehensively and efficiently cure this disease, understanding the complete mechanism underlying NAFLD is vital [15].

To develop effective drugs and treatments for NAFLD disease, several biological processes such as hepatic fat and oxidative stress which originate from the imbalance between antioxidant defenses and reactive oxygen species generation, are under consideration [16-20]. Studies have revealed promising insights into the mechanisms underlying NAFLD [21, 22]. Advances in molecular biology techniques and next-generation sequencing, such as exome, genome, and transcriptome sequencing, are enabling researchers to investigate the mechanism of NAFLD [23, 24] and can lead to the identification of novel biodiagnostic markers and innovative drug candidates for the management of NAFLD.

In the present study, we have carried out 3-D structure prediction, molecular docking, dynamics, and simulation of the protein-ligand complex with several metrics such as the root mean square fluctuation (RMSF) and root mean square deviation (RMSD). The simulation that the compounds PNPLA3-71448940, PNPLA3-10033935, and PNPLA3-5748394 were stable at 400ns, while compound PNPLA3-71448940 was found to be most stable because of its strong hydrogen bonding and other parameters.

## 2 MATERIALS AND METHODS

The flowchart of the adopted computer-aided drug design (CADD) is given in Fig. (**1**). The details of the methodology are as follows:

### 2.1 Target Identification, Structure Prediction and Analysis

The Patatin-like phospholipase domain-containing protein 3 (PLPL3) has been linked to detrimental metabolic outcomes, such as insulin resistance and dyslipidemia [25-27]. In certain people, hepatic steatosis also induces steatohepatitis, an inflammatory response in the liver that can lead to cirrhosis and liver cancer. Therefore, PLPL3 proteins were selected as potential drug targets. Moreover, RNA seq data relevant to NFLD were also obtained from the GEO Omnibus dataset and the expression of PLPL3 genes with a *p* < 0.01 was considered significant. These data are available on the NCBI GEO repository (GSE135251) [28].

### 2.2 Homology Modeling and Protein Properties

We obtained the three-dimensional structures of PLPL3 druggable protein targets for our molecular docking experiments. An *in silico* comprehensive study was carried out to retrieve NAFLD-associated proteins from the UniProt, NCBI, and EBI databases, laying the foundation for a study of the molecular landscape of this disease [29, 30]. BLASTP analysis was carried out to search the template sequence for modeling using homology searches against the Protein Data Bank (PDB) [31]. The SAVES v5.0 server was used to assess and design the protein models. Five different tools for evaluating model results are included in SAVES v5.0 server such as 3D, ERRAT, PROVE, PROCHECK, and WHATCHECK verification [32, 33]. DruLito and SwissADME were utilized to assess the physical and chemical characteristics of the ligands. These platforms employ computations grounded in diverse drug-likeness criteria, including the MDDR-like rule, Lipinski's rule, the Ghose filter, the Veber rule, the CMC-50-like rule, the Quantitative Estimate of Druglikeness (QED), and the BBB-rule.

### 2.3 Molecular Docking Analysis

Molecular docking was carried out against the active site of the query protein with the provided ligand using AutoDock software. The ligand molecule exhibiting the minimum free energy was selected as the foundational molecule for designing the in-silico structure of a drug molecule targeting NAFLD. For the large-scale molecular docking of drugs to protein targets, computational pipelines such as PyMOL, AutoDock Vina, and Discovery Studio were used [34-40]. The docked compounds, based on their binding affinities, were selected for further protein–ligand complex analysis, which revealed the interactions between the receptor and inhibitor along with the protein and its binding sites. Moreover, 1000 molecules were considered and ligand formats included SDF (Structure Data File) and MOL2. These formats were converted to PDBQT using Open Babel [32, 34] and AutoDockTools to ensure compatibility with AutoDock Vina. This involved preparing the ligands by adding hydrogen atoms, defining rotatable bonds, and assigning Gasteiger charges [35], which are crucial steps to accurately simulate the docking process and interactions within the target protein's active site. The details of the docking analysis are as follows Dimension: x-dimension:40, y-dimension:40, z-dimension: 40, center gride box: x center: -26.599: y center: 12.599: z center: 58.966, residues considered for grid generation: Ile148, Asp166, Ser47, and Met148 [40, 41].

### 2.4 Protein-ligand Interactions and Pharmacophore Generation

To design and develop new drugs, the pharmacophore models and protein-ligand interactions are crucial [42, 43]. The LIGPLOT software was utilized to identify protein ligand interactions which are helpful for understanding interactions between ligands and receptors, and it is vital to obtain insights into the structural requirements for therapeutic potential and effective binding [44]. The Ligand Scout software was used for pharmacophore modeling [45].

### 2.5 Toxicity Analysis

ADMET stands for absorption, distribution, metabolism, elimination, and toxicity (ADMET). The potential efficiency of the drug compound is ensured by modeling following ADMET, which determines the effectiveness, metabolic behavior, uptake, safety, and elimination of a drug. For these properties, the compound with the lowest binding affinity and the greatest number of interactions was selected [46, 47] following the prediction of ADMET using the AdmetSAR tool [48-50].

### 2.6 Molecular Dynamics Simulation and Principal Component Analysis

The prediction of the dynamic properties and reliability of compounds at the atomic level is vital and molecular dynamics (MD) simulations play a key role in elucidating the dynamic motion of individual atoms within proteins and other molecular systems [51, 52]. The simulated paths were thoroughly investigated using Desmond [53, 54]. The chosen lead molecule was then subjected to MD simulations to gain complete knowledge of the smallest alterations and variances within the receptor-ligand system [55, 56]. To perform the simulation, an OPLS_2005 force field was used. Intermolecular Interaction Potential 3 Points Transferable TIP3P, the orthorhombic box solvent model included in the simulation setup, and counterions were included to maintain the equilibrium of the models [57]. In the simulation of physiological conditions, 0.15 M sodium chloride was used. In addition, during the simulation process, NPT ensembles were maintained at 310 K and 1 atm of pressure and trajectories were kept at 100 ps intervals. Tracking of the ligand over time and the RMSD of the protein further confirmed the stability of the simulation. The next step was to perform PCA using the Bio3D R package and determine the stability of the potential drug targets from the simulation data [58, 59].

### 2.7 MM-GBSA: Molecular Mechanics and Generalized Born Surface Area Calculations

Prime module of MM-GBSA utilized to identify the binding free energy of selected docked complexes during the simulation of SOD1 complex with Scytonine and Raocyclamide_A. For free binding energy calculations, VSGB solvent model, search techniques of rotamer and OPLS 2005 force field were employed. The frames of MD trajectory after running of simulation at interim of 10ns followed equation 1 that was used for free energy calculations,


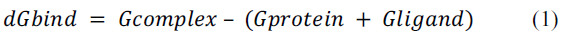


Where, dGbind = binding free energy, Gligand = free energy of the ligand, Gprotein = free energy of the target protein and Gcomplex = free energy of the complex.

## 3 RESULTS

### 3.1 Protein Selection and Structural Refinement

**Fig. (2):**
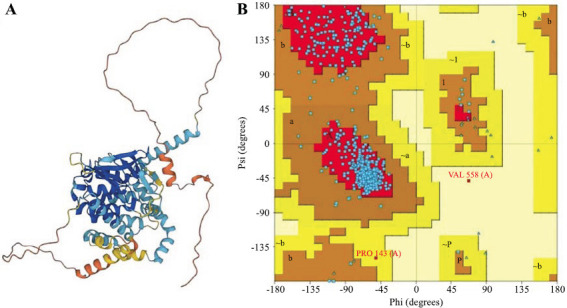
**A**). Three-dimensional structure of protein, **B**). Ramachandran Plot, showing different regions of protein structure.

**Fig. (3):**
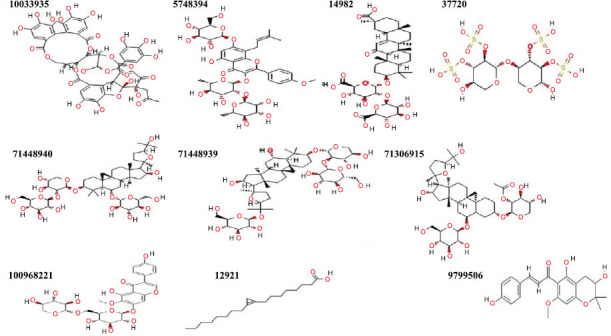
Identification of NAFLD inhibitors using different chemical information sources such as PubChem and ZINC.

For this purpose, PNPLA3 gene, which syntheses a protein called adiponutrin, was selected for further investigation to determine its 3-D structure. Since the protein structure was determined to have an e-value of 0, identity> 80% and region 1-481 were found, and a 3-D model of the protein named AF-Q9NST1-F1 involved in NAFLD (PNPLA3) was retrieved in PDB format (Fig. **2A**) and 3-D structure of PNPLA3 was designed by MODELLER. Through graphical representation obtained using Rampage, analysis of the Ramachandran plot indicated that 99.2% of the residues were in the most favored region of the refined structure (Fig. **2**). Moreover, the overall quality factor in the ERRAT program escalated to 92.5%, which shows a good quality structure, as depicted in Fig. (**2**).

Moreover, inhibitors against NAFLD were identified using different chemical information sources such as PubChem and ZINC as shown in Fig. (**3**). The compound ID and details of these inhibitors are also given in Table **1**. For example, CID 10033935 composed of ellagitannin, CID 5748394 to epimedin B are among the ten inhibitors that were selected from 1000 compounds of phytochemical library molecules. These are those selected during Lipinski and Veber filtering (such as logP > 5, H-Bond acceptors > 10, PSA < 140, RB < 10, Molecular Weight > 500, H-Bond Donors > 5).

### 3.2 Molecular Docking

Molecular docking is a process that determines the preferred coordination between two molecules when molecules are in close proximity and generates the interactions among them leading to the formation of complexes. In our study, the primary goal of molecular docking was to determine the optimal interactions between ligand and protein molecules. The results of ten docked compounds are shown (Table **1**). After docking analysis, the complex with the lowest binding energy was considered the “best match”. For example, the compound ID 10033935 (ellagitannin) showed an S Score of 18.962, HDB 13, HAB 22, and a minimum E-score -17.2658, which were better than those of the other compounds, as described in Table **1**.

### 3.3 Protein Ligand-interaction

Analysis of protein and ligand interactions was carried out using LIGPOT. To obtain the lead compound based on the docking and interaction results, the main purpose of this tool was to identify interactions between the ligands and their environmental residues. The results of protein-ligand interactions for compound ID 10033935 (ellagitannin) are shown in Fig. (**4**).

This ligand has different characteristics, such as different ligand bonding, nonligand bonding and hydrogen bonding distances. Green indicates different amino acids involved in protein interactions, the dotted green line represents the bonding distance of residues, the black circle represents ligand bonding, and the blue circle represents nonligand bonding of residues. The best protein‒ligand interaction was that of the ellagitannin compound (10033935), which shows a bonding distance within the range of 3 angstroms and interacts with these residues (Glu205, Glu206, Asn710, Ser630, and Tyr547).

### 3.4 Pharmacophore Modeling

Pharmacophore models were generated for NAFLD drugs. These pharmacophore models were designed to better understand interactions between receptors and ligands. Using the Ligand Scout software, the top five compounds shown in Fig. (**5**) were selected.

### 3.5 Toxicity Analysis and Lead Compounds Identification

ADME/Tox is used to describe the absorption, distribution, metabolism, excretion and toxicity of drugs. Particularly in the preclinical stages, the *in silico* ADME/Tox profile is a helpful tool for predicting the pharmacological and toxicological characteristics of drug candidates. The toxicity of ellagitannin was assessed using the AdmetSAR web server. The results revealed that ellagitannin met the structural provisions for drug development by following Lipinski's Rule of Five. Such as ellagitannin has high adsorption and dispersion values, demonstrating that it can easily pass through cell walls. The potential of ellagitannin as a lead molecule for NAFLD treatment is limited by its lack of toxicity, *i.e*., absolute values less than 100 and favorable Log PS, Log BB, and log Kp values. So, based on all the above analyses, results revealed that compound Ellagitannin (10033935) was identified as the most active of all the tested compounds after toxicity analysis.

### 3.6 A Molecular Dynamics Simulation

**Fig. (6):**
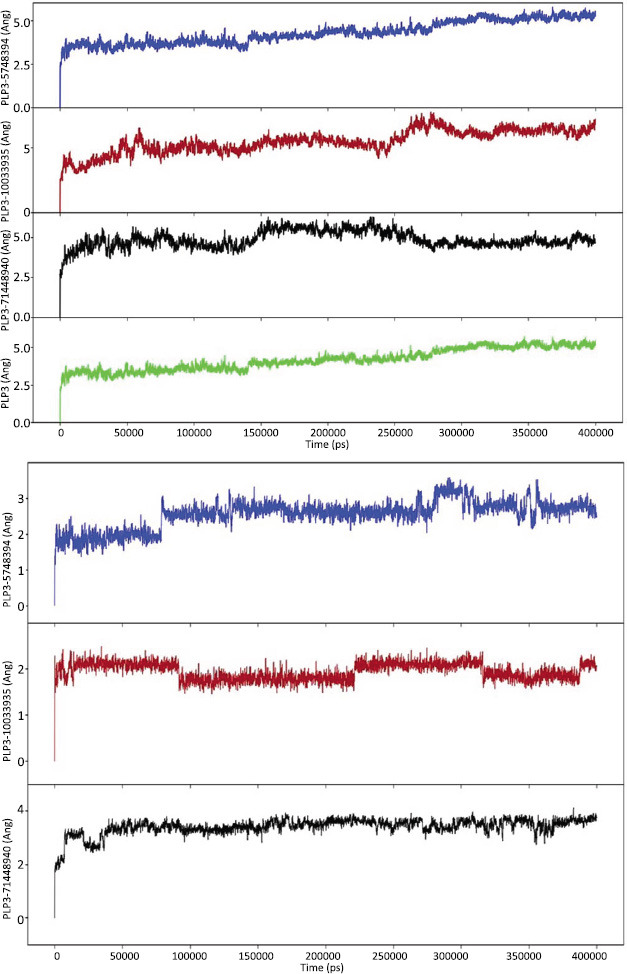
**A**). The RMSD of the carbon alpha atoms of the proteins complexed with ligands are shown with respect to time. The black color represents PNPLA3-71448940, the red color represents PNPLA3-10033935, and the blue color represents the PNPLA3-5748394 complex proteins. **B**). The RMSD of ligand atoms complexed with drug targets. The black color represents PNPLA3-71448940, the red color represents PNPLA3-10033935, and the blue color represents the complexed ligand of PNPLA3-5748394.

A molecular dynamics simulation lasting 400 ns was run on the protein target and its optimal chemical compound. The simulated paths were thoroughly investigated using Desmond. To evaluate the dynamics and structural stability of the protein-ligand complex, a number of metrics were produced throughout the simulation, such as RMSF and RMSD (Figs. **6A** and **B**). MD trajectory analysis was carried out to examine the complicated connections during the simulation. Fig. (**6**) shows how the RMSD values of the carbon alpha atoms in ligand-bound proteins vary with time. The NAFLD protein reached stability at 10 ns, as illustrated by the RMSD plot. The RMSD values then varied as the simulation progressed but remained constant for all three complexes. The protein RMSD increased significantly over the times but then stabilized again, even though there was minimal overall structural change, signifying a stable structure. The constant RMSD of the ligands in the graph indicated that they were stable throughout the trial. In compounds PNPLA3-10033935 and PNPLA3-5748394, there were discernible variations in the RMSD, which might indicate a modification in the binding mode. After that, when equilibrium was reached in the last 400 ns of the experiment, the ligand RMSD remained constant. As a result, the ligand RMSD reached equilibrium at 10 ns and remained steady and dependable throughout the experiment (Figs. **6A** and **B**).

**Fig. (7):**
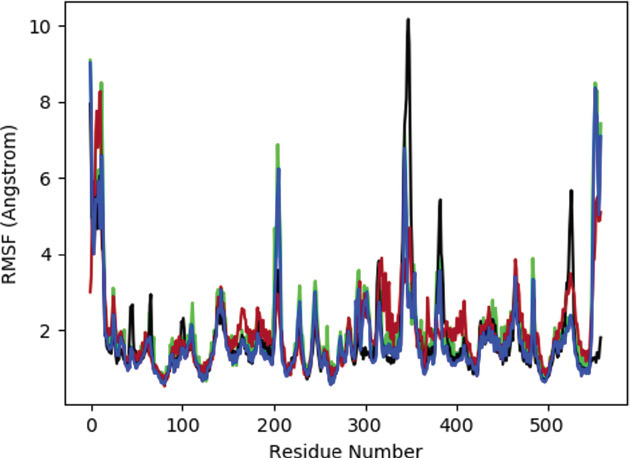
Residue wise root mean square fluctuations for each of the three complexed proteins with linked ligands. The black color represents PNPLA3-71448940, the red color represents PNPLA3-10033935, the blue color represents the PNPLA3-5748394 complex protein and green shows apoprotein.

The correlation between different protein designs and ligands is demonstrated by the RMSF data in Fig. (**6**). Based on the MD progression data, the peaks in Fig. (**3**) align with the loop and the N and C termini. Reduced RMSF values at binding site residues imply that the protein and ligand are permanently attached (Fig. **7**). Strings and helices became important secondary structural elements (SSEs) throughout the modeling phase. The distribution of these structural elements by residue during the course of the simulation is shown in the graph.

**Fig. (8):**
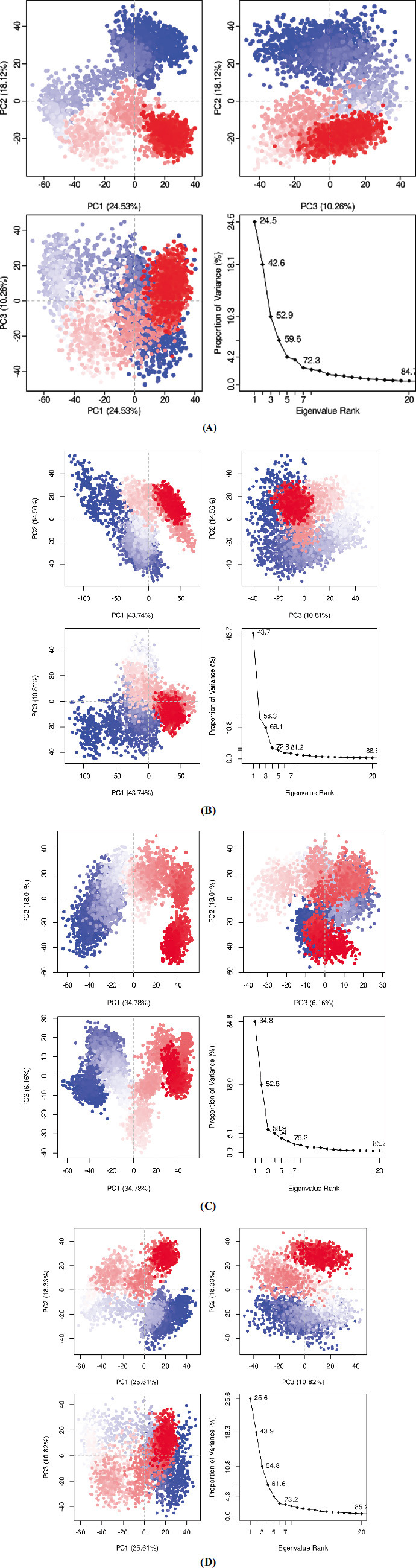
A Principal Component Analysis was performed, and the eigenvalues were plotted against the percentage of variance for three distinct sections. The cumulative variances attributed to variations in PC1, PC2, and PC3 were determined. **A**) For the PLPL3-71448940 complex, these values were 24.53%, 18.12%, and 10.26% respectively. **B**) Similarly, for the PLPL3-10033935 complex, the cumulative variances for PC1, PC2, and PC3 were found to be 43.74%, 14.56%, and 10.81%, respectively. **C**) For PLPL3-5748394 complex, the cumulative variances for PC1, PC2, and PC3 were found to be 34.78%, 18.01%, and 6.16%, respectively. **D**) For Apo protein PC1, PC2, and PC3 were found to be 25.61%, 18.33%, and 10.82%, respectively.

**Fig. (9):**
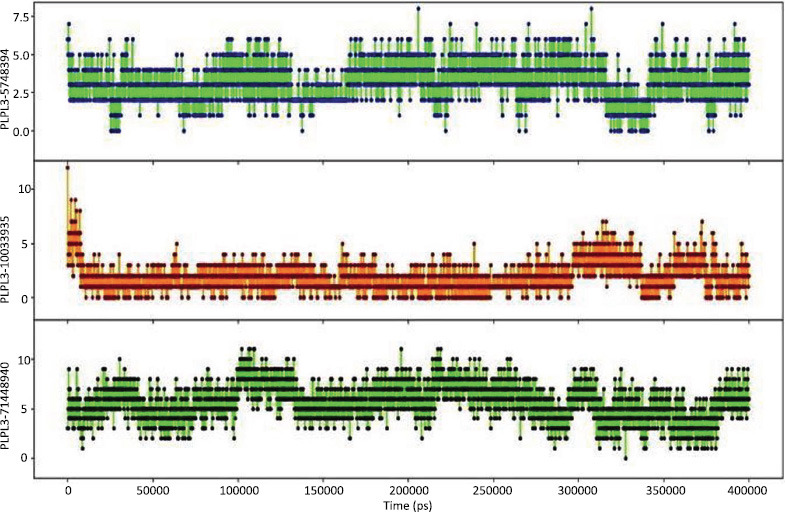
The interaction between protein and ligands was tracked continuously during the simulation for all three complexes. The black color represents PNPLA3-71448940, red shows PNPLA3-10033935, and blue represents PNPLA3-5748394 complexes.

In this work, the investigation of protein dynamics was aided by the application of Principal component analysis (PCA). Following a detailed analysis of the collective trajectory motions during molecular dynamics (MD) simulations, the first 20 modes of motion in each complex (PNPLA3-71448940, PNPLA3-10033935, and PNPLA3-5748394) were plotted against the eigenvector index (eigenmode) to find a stable and consistent pattern. Larger eigenvectors were discovered to be important regulators of the total mobility of the target protein in the context of simulation research. The greater eigenvalues for the top five eigenvectors in the systems under examination differed from those of the other eigenvectors (24.5% to 72.3% for PNPLA3-71448940, 43.7% to 81.2% for PNPLA3-10033935, and 34.8% to 75.2% for PNPLA3-5748394). By highlighting hydrogen bonds as the main interactions discovered by MD analysis, Fig. (**5**) emphasizes the significance of receptor-ligand interactions (Fig. **8**). The most important residue bonding sites in the PNPLA3-71448940 complex are GLU_362, LYS_363, LYS_410, GLN_411, THR_412, TYR_422, and LEU_548. GLN_263, GLN_266, and GLU_440 are the most significant hydrogen bonds in the complex PNPLA3-10033935. LYS_410, GLN_411, and GLU_543 are the most important residues at the bonding sites in PNPLA3-5748394 (Fig. **9**).

We used MM-GBSA calculations to analyze protein-ligand complexes across a 400 ns trajectory, and we observed substantial changes in the ligands' binding affinities. With an average total energy of -88.3608 kcal/mol, ligand 71448940 had the greatest binding affinity and suggested a highly stable association. With an average energy of -58.036 kcal/mol, ligand 10033935 demonstrated a lower binding affinity than ligand 5748394, which demonstrated even weaker binding with an average energy of -55.1045 kcal/mol (Table **2**). Tables **S1-S3** provide comprehensive MM-GBSA findings, illustrating the binding affinities over time of each ligand. The ligand 71448940 has the strongest binding to the protein complex PLPL3, indicating that it is a worthy subject for more investigation. In contrast, interactions between ligands 1033935 and 5748394 are weaker, suggesting that they may be less efficient. Future research and the creation of possible medicinal medicines are guided by these findings.

## 4 DISCUSSION

Due to the narrow range of available therapies for NAFLD, it has become a common and increasingly disabling disorder for which substantial therapeutic options are available. The major cause of NAFLD has emerged as the sedentary lifestyle globally [60, 61]. A comprehensive strategy is needed, due to the complex nature of NAFLD, to reassess daily activity patterns, dietary practices, and social standards [62-64]. Using MODELLER, steady 3-D models of the selected druggable protein targets were generated before molecular docking experiments. The results of the docked compounds substantiated our findings according to reported studies [65]. This step laid the foundation for designing computer-aided drugs, a contemporary approach to the search for more effective medications [66, 67]. Molecular docking analysis was carried out by using the Autodock software on a targeted library of phytochemical compounds. Based on a notable E-Score of -18.9622 and an RMSD of less than 3, the most promising potential candidate was Ellagitannin, demonstrating that it docked well in the active region of the protein. The selection of appropriate docked compounds was based on Lipinski's criteria [68]. Moreover, Astragaloside VI, Glycyrrhizin, Epimedin C, and Pentosan Polysulfate also exhibited promising RMSD and S-Score values. The assessment of the S-score and RMSD was corroborated, as described by Castro-Alvarez *et al.* [38].

According to previous studies, LIGPLOT revealed the most promising molecular interactions between proteins and ligands with bond lengths of less than 3 angstrom units [68, 69]. The most appropriate ligand that had very close interactions with key protein residues, such as Glu20, Asn70, Glu206, Ser630, and Tyr547, was ellagitannin. In further studies, Ligand Scout Software revealed and generated the top five pharmacophore models, namely, Ellagitannin, Pentosan Polysulfate, Epimedin C, Glycyrrhizin acid, and Astragaloside VI. As described previously by de Souza Neto *et al.* [40], the best pharmacophore model is characterized by a hydrogen bond donor (HBD), hydrogen bond acceptor (HBA), hydrophobic-centroids (HYP), and aromatic ring (AR), and ellagitannin displayed higher performance for each of these pharmacophore features. Safety evaluation and risk assessment of the potential lead compound are important [69-71], and the AdmetSAR tool confirmed that compounds met the structural provisions for drug development by following Lipinski's Rule of Five. For example, potential compounds have high adsorption and dispersion values, demonstrating that they can easily pass through cell walls. The potential of ellagitannin as a lead molecule for NAFLD treatment is limited by its lack of toxicity, *i.e*., absolute values less than 100 and favorable Log PS, Log BB, and log Kp values, which are similar to the findings of Janin *et al*. [41].

Overall, Ellagitannin was identified as a potential druggable target. Ellagitannins and their metabolites, including ellagic acid and urolithin, have the potential to treat NAFLD through several key mechanisms. These compounds exert antioxidant and anti-inflammatory effects, regulate lipid metabolism, activate AMPK, improve insulin sensitivity, reduce fibrosis, and influence gut microbiota. Through these diverse actions, ellagitannins effectively target the main pathological features of NAFLD, including lipid accumulation, oxidative stress, inflammation, insulin resistance, and liver fibrosis [5, 69-71]. Ellagitannin has shown potential as a treatment for NAFLD through extensive *in silico* analyses, including molecular docking and dynamic simulations. It demonstrated strong protein-ligand interactions, stability, good drug-like properties, and low toxicity [72]. These findings suggest that ellagitannin could be a valuable candidate for developing new NAFLD treatments, but further clinical research is needed. Currently, managing NAFLD involves maintaining a healthy weight, balanced diet, regular exercise, limiting alcohol consumption, and controlling medical conditions, which can significantly reduce the risk of the disease. Considering the promising potential of ellagitannin, the stability of the top 10 compounds was analyzed and the most important residue bonding sites in the PNPLA3-71448940 complex were GLU_362, LYS_363, LYS_410, GLN_411, THR_412, TYR_422, and LEU_548. GLN_263, GLN_266. The histogram that is displayed shows the total number of hydrogen bonds, ionic connections, hydrophobic interactions, and water bridges that occurred between the protein and ligand throughout the experiment.

Similarly, molecular dynamics and simulation revealed promising novel potential compounds such as both PNPLA3-10033935 (Ellagitannin), PNPLA3-71448940, and PNPLA3-5748394 complex proteins at 400ns with very slight variation. Moreover, there was evidence that a protein was bound to a ligand during the experiment. The histogram that is displayed shows the total number of ionic connections, hydrogen bonds, hydrophobic interactions, and water bridges that occurred between the protein and ligand throughout the experiment, as shown in Fig (**9**).

In a number of crucial areas, NAFLD has advanced significantly and research has shown how important it is to precisely diagnose and treat NAFLD. Nevertheless, there is a dearth of authorized pharmacotherapies, which makes further clinical research and creative approaches to therapy necessary. Furthermore, it is important to review and refine screening techniques for individuals who are at risk to facilitate early intervention and avoid disease [73].

Ellagitannins have shown potential in influencing the molecular mechanisms associated with NAFLD through various pathways. They exhibit strong antioxidant properties, which help reduce oxidative stress in liver cells, a significant factor in NAFLD progression. Additionally, Ellagitannins modulate inflammatory pathways by inhibiting pro-inflammatory cytokines and enzymes such as NF-κB and COX-2, thereby reducing liver inflammation. Ellagitannins also play a role in regulating lipid metabolism by affecting genes involved in lipid synthesis and degradation, leading to decreased hepatic lipid accumulation. They improve insulin sensitivity, which is crucial for managing NAFLD, as enhanced insulin sensitivity helps mitigate the risk of hepatic steatosis. Currently, treatments for NAFLD involve pharmacological therapies, lifestyle modifications, and surgical options and Oral diabetes medicines like metformin and sitagliptin. When we compare these treatments with Ellagitannin which is metabolized into ellagic acid (EA), it shows promise as a potential treatment for non-alcoholic fatty liver disease (NAFLD) [69, 70]. In the future, this drug will protect against NAFLD and become more effective than other treatments for non-alcoholic fatty liver disease.

## CONCLUSION

An *in silico* approach comprising different steps has been applied in the current research to characterize and identify potential drug targets and suggest drugs for NAFLD. The use of computational tools and databases helped us to better understand the cause of disease and to identify better drugs against specific proteins. Ellagitannin (10033935) has the lowest toxicity, making it an excellent target for the development of therapeutics. The molecular dynamics and structural stability of the protein‒ligand complex were evaluated with several metrics, namely, the RMSF and RMSD, and the results revealed the stability of the PNPLA3-71448940, PNPLA3-10033935 and PNPLA3-5748394 complex proteins at 400 ns. We concluded that elagitannin could be a potential druggable target with a favorable therapeutic profile for the treatment of NAFLD, a disorder for which there are currently no proven therapies.

## Figures and Tables

**Fig. (1):**
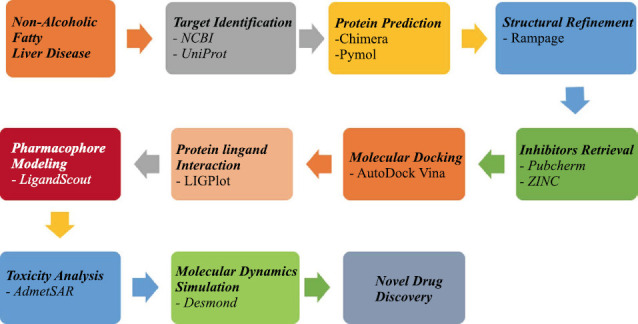
Flowchart of adopted computer-aided drug design (CADD) methodology.

**Fig. (4):**
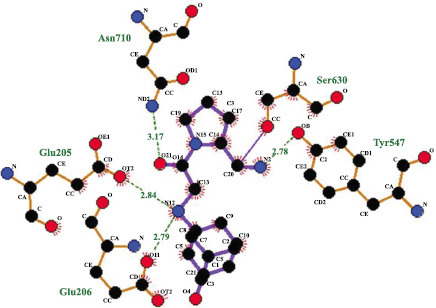
Schematic diagram of protein-ligand interaction of ellagitannin (10033935).

**Fig. (5):**
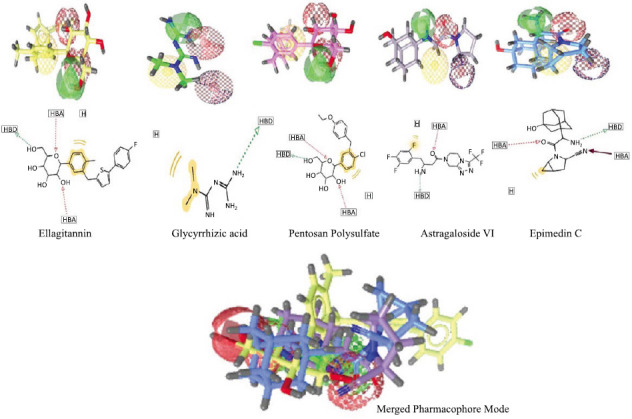
Pharmacophore models: **A.** Ellagitannin (10033935), **B.** Glycyrrhizic acid (5748394), **C.** Pentosan Polysulfate (14982), **D.** Astragaloside VI (37720), **E.** Epimedin C (71448940) and **F.** Merged Pharmacophore Mode. These pharmacophore model consists of four pharmacophore features namely, hydrogen bond acceptor (HBA), hydrogen bond donor (HBD), hydrophobic (HYP) centroids and aromatic-ring (AR).

**Table 1:** Docking results of the compounds.

**S. No.**	**Compound ID**	**Score**	**Rsmd Value**	**HDB**	**HAB**	**E Score**
1	10033935 (Ellagitannin)	18.9622	2.38	13	22	-17.266
2	5748394 (Epimedin B)	17.9935	1.7973	5	18	-14.778
3	14982 (Glycyrrhizic acid)	16.1799	1.8456	4	16	-12.244
4	37720(Pentosan Polysulfate)	15.4122	1.6137	2	21	-14.034
5	71448940(Astragaloside VI)	14.0454	2.2557	10	19	-14.255
6	71448939(Astragaloside V)	13.5715	2.4136	8	19	-11.813
7	71306915(Astragaloside-II	12.4475	2.4291	6	14	-11.922
8	100968221(Tectorigenin 7)	11.1226	1.9912	6	14	-13.463
9	12921(Sterculic acid)	10.102	1.8297	0	2	-10.919
10	9799506(xanthohumol B)	-9.0707	1.647	3	6	11.5522

**Table 2:** The MM-GBSA calculation of binding energy of ligands with PLPL3 from MD simulation trajectories performed every 50 ns. (Detailed analysis of MM-GBSA is submitted in Tables S1-S3).

**Time**	**dG_bind_** **PLP3-71448940**	**dG_bind_** **PLPL3-10033935**	**dG_bind_** **PLPL3-5748394**
0 ns	-121.41	-64.1181	-62.465
50 ns	-83.7835	-55.857	-52.0871
100 ns	-110.968	-69.0734	-55.1666
150 ns	-99.1254	-48.5258	-49.1461
200 ns	-59.5879	-58.2635	-49.5784
250 ns	-71.378	-53.7135	-54.4159
300 ns	-81.7287	-50.9219	-60.3105
350 ns	-74.7636	-63.6527	-53.3616
400 ns	-92.5019	-58.1998	-59.4091
Average dG_bind_	-88.3608	-58.0362	-55.1045

## Data Availability

The protein sequence and data used in this study are available within the article.

## LIST OF ABBREVIATIONS

NAFLD: Nonalcoholic Fatty Liver Disease
RMSF: Root Mean Square Fluctuation
T2D: Type 2 Diabetes
