# Supplementary material for: Decoding the Knacks of Ellagitannin Lead Compounds to Treat Nonalcoholic Fatty Liver Disease using Computer-aided Drug Designing
Source: Curr Comput Aided Drug Des. 2024 Oct 7;21(8):1108–22. doi: 10.2174/0115734099325555240927054614 (PMC12728524; doi:10.2174/0115734099325555240927054614)
Supplement: Supplementary file 1 [file CCADD-21-8-1108_SD1.pdf]

SUPPLEMENTARY MATERIAL

Decoding the Knacks of Ellagitannin Lead Compounds to Treat Nonalcoholic Fatty Liver Disease using Computer-aided Drug Designing

Hina Shahid[1], Muhammad Ibrahim[2], Wadi B Alonazi[3] and Zhanyou Chi[1,*]

[1]MOE Key Laboratory of Bio-Intelligent Manufacturing, School of Bioengineering, Dalian University of Technology, Dalian, Liaoning, China; [2]State Key Laboratory of Rice Biology and Breeding, Key Laboratory of Molecular Biology of Crop Pathogens and Insects, Institute of Biotechnology, Zhejiang University, Hangzhou 310058, China; [3]Health Administration Department, College of Business Administration, King Saud University, Riyadh, Saudi Arabia
